# “Swallowing these drugs every day, you get tired”: a mixed-methods study to identify Barriers and facilitators to retention and HIV Viral Load suppression among the Adolescents living with HIV in TASO Mbale and TASO Soroti centers of excellence

**DOI:** 10.21203/rs.3.rs-3863602/v1

**Published:** 2024-01-17

**Authors:** Bonniface Oryokot, Andrew Kazibwe, Abraham Ignatius Oluka, David Kagimu, Baker Bakashaba, Saadick Ssentongo, Twaha Mafabi, Charles Odoi, Abubaker Kawuba, Yunus Miya, Bernard Michael Etukoit, Kenneth Mugisha, Eleanor Namusoke-Magongo

**Affiliations:** AIDS Information Centre; The AIDS Support Organization; AIDS Information Centre; The AIDS Support Organization; AIDS Information Centre; AIDS Information Centre; The AIDS Support Organization; The AIDS Support Organization; The AIDS Support Organization; The AIDS Support Organization; The AIDS Support Organization; AIDS Information Centre; Ministry of Health

**Keywords:** Adolescents, HIV, Viral Load, Retention, Barriers, Facilitators

## Abstract

**Background:**

Adolescents aged 10–19, living with HIV (ALHIV) lag behind in attaining optimal viral load suppression (VLS) rates and retention in care, an important impediment to reaching epidemic control. This study aimed to identify barriers and facilitators to both VLS and retention among in the sub-population who seek care from TASO Mbale and TASO Soroti centers of excellence, to facilitate adaptation of the operation triple zero in the setting.

**Methods:**

We used a mixed methods approach, extracting secondary data on ALHIV who were active in care during April-June 2022 quarter to determine one year retention in care. Analysis was done in STATA Corp, 15.0. We used logistic regression to determine associated factors and adjusted odds ratio (aOR) to report level of predictability, using 95% confidence interval (CI) and P<0.05 for statistical significance. For qualitative component, purposive sampling of 59 respondents was done. Focused group discussions, key informant interviews, and in-depth interviews were used to collect data. Thematic content analysis was done using Atlas ti.

**Results:**

There were 533 ALHIV, with 12-month retention rate of 95.9% and VLS rate of 74.9%. Predictors for good VLS included good adherence [aOR:95%CI 0.066(0.0115, 0.38) P=0.02], being on first line treatment [aOR:95%CI 0.242 (0.0873,0.6724) P=0.006]. For retention, they include being a school going [aOR:95%CI 0.148(0.024,0.9218) P=0.041], multi month dispensing aOR:95%CI 32.6287(5.1446,206.9404) P<0.001, OVC enrolment aOR:95%CI 0.2625(0.083, 0.83) P=0.023]. Meanwhile key barriers included: individual ones such as internal stigma, lack of transport and treatment/drug fatigue; facility-level such as prolonged waiting time and lack of social activities; community level include stigma and discrimination, inadequate social support and food shortage. In terms of facilitators, individual level ones include good adherence and knowledge of one’s HIV status; facility-level such as provision of adolescent friendly services and community-level such as social support and decent nutrition.

**Conclusions:**

VLS rate was sub-optimal mainly due to poor adherence. HIV programs could utilize the barriers and facilitators identified to improve VLS. Conversely, retention rate at one year was good, likely due to provision of adolescent friendly health services. ALHIV and their caregivers need to be empowered to sustain retention and improve VLS.

**Contributions to science:**

By accentuating the barriers and facilitators to retention and VLS among the ALHIV, we ensure HIV programs continue to prioritize effective interventions and discard others as the epidemic evolves. To this, our findings strategically validate the effectiveness of provision of adolescent friendly services and client-centered care in attaining good retention rate.

Secondly, being a mixed-methods study, complementarily adds value to the existing body of knowledge on barriers and facilitators while reminding programmers that VLS remains sub-optimal and more efforts are necessary.

Finally, different stakeholders could use our findings to advocate for more resources to address some of the barriers such as food shortage, empowerment of ALHIV and caregivers and strengthening skilling programs for ALHIV, especially the out-of-school.

## Introduction

One of the global aspirations to efinding HIV/AIDS as a public health problem is ensuring that 95% of all people living with HIV (PLHIV) across all populations and age groups attain and sustain HIV viral load suppression (VLS). This requires that PLHIV are identified, initiated on antiretroviral therapy (ART), retained in care and remain adherent. To this, there is notable progress with five countries including Eswatini, Botswana, Tanzania, Zimbabwe and Rwanda already achieved the milestone in the general population [1]. However, the adolescents (aged 10–19 years) living with HIV (ALHIV) remain disproportionately behind [2].

In 2022, 27,000 (about 4% of global AIDS-related mortality) AIDS-related deaths occurred among the 1.65 million ALHIV, majorly residents in sub-Saharan Africa [3]. Furthermore, The Joint United Nations Program Against HIV/AIDS (UNAIDS) estimates, indicated that the global VLS in the sub-population was at 46% during the same year. A recent systematic review in SSA, reported a 55% VLS among the ALHIV and 65% adherence [4]. Similarly, In Uganda,. Routine programmatic data indicate that by the end of June 2022, retention rate at one year stood at 65% and VLS rate at 71%., both far below the 95% global targets.

Barriers to optimal VLS identified include; difficulties fifinding transport money, unfriendly health settings, drug stock outs, prolonged waiting time, unfavorable school timetable, non-disclosure of HIV status and drug side effects [4–10]. Conversely, identified enablers include reliable stock-levels of drugs in the facilities, good attitude of health-workers, availability of transport money, social/family support, and positive peer influence [4, 7, 8]. To counteract the barriers while enhancing the enablers, interventions implemented in different settings across Uganda. include; treatment optimization with dolutegravir (DTG)-based regimens, peer-driven approaches such as the Youth and Adolescent Peer supporters (YAPS), and differentiated service delivery models including multi-month dispensing [11–13].

Whereas many barriers and facilitators to retention and VLS are known, both retention and VLS remain sub-optimal across several settings in Uganda, including The AIDS Support Organization (TASO) Uganda. Additionally, the factors tend to vary from one setting to another, possibly justifying the persistent sub-optimal levels of the two treatment outcomes despite the interventions so far implemented. Thus, we proposed to adapt the implementation of operation triple zero (OTZ) [14] model in the TASO setting to close these gaps. As part of the implementation strategy, we identified barriers and facilitators to both retention and VLS to facilitate adaptation of OTZ in the setting.

## Methodology

### Study Design

A mixed-method study (explanatory sequential) was preferred to comprehensively elicit barriers and facilitators to retention and VLS.

### Study Setting

We conducted the study in TASO Mbale and TASO Soroti centers of excellence (COEs). The two TASO centers are located in eastern Uganda, approximately 223 kilometers and 323 kilometers east of Kampala capital city respectively. Both COEs provide comprehensive HIV services such as prevention, care, treatment and support to more than 13300 PLHIV including 583 ALHIV as at the end of September 2022. The COEs cover a radius of 75kilometers of catchment area of operation, reaching a combined total of 20 districts. In terms of adolescent services, both facilities implement the YAPS model, in line with the ministry of health standards. The YAPS is an adaptation of the community adolescent treatment supporter (CATS) of the Zvandiri intervention in Zimbabwe [15, 16]. The YAPS are typically young people living with HIV, have overcome internal stigma, can constructively write and speak in English language [13]. They received basic training in peer-to-peer work before deployment. Thus, expected to provide high-quality HIV/TB services that are age-appropriate [11]. Further, viral load tests are the standard measure of treatment response and plasma samples are shipped to the central public health laboratory for analysis. Viral load copies of ≤200/mL were considered suppressed while those above, non-suppressed in line with the national guidelines [13]. Both facilities utilized only plasma samples for VL tests. Finally, it is worth noting that the two COEs offer a similar model of adolescent care, as described by Asire et al., [17]. TASO Mbale COE conducts an independent adolescent clinic, twice weekly, ensuring that the adolescents are spread out more evenly on the clinic visits. This enables the health workers to sufficiently interact with the ALHIV. On the other hand, TASO Soroti operates an independent adolescent clinic that is run monthly. While this enables adolescents to catch-up since many attend the clinics, it also has the potential of overwhelming health workers with work, given the size of the attendances.

### Study Population

We considered ALHIV who were active in care in the April-June 2022 quarter. In addition, caregivers and health workers including peers, were also engaged to provide qualitative insights.

### Inclusion Criteria

All ALHIV active in the April-June 2022 quarter, and aged 10–17 years, to allow for complete follow-up of the cohort for at least one year.

### Exclusion Criteria

ALHIV aged 18 years and above were excluded as these would have transitioned within 12 months of implementation. In addition, those with incomplete information on key variables such as ART status and VL were excluded.

### Sample size and Sampling Procedure

#### For Quantitative Component

We used a census sampling technique, enrolling all the eligible ALHIV into the study.

#### Qualitative component

A purposive sampling approach was preferred. A total of 59 ALHIV with suppressed and non-suppressed VL were purposely mobilized together with their caregivers and health workers to participate in the study.

#### Study Outcomes

##### HIV Viral Load Suppression

We adopted a standard ministry of health definition of VLS; copies of ≤200/mL considered to be suppressed and those above, non-suppressed. We considered any VL test done within the previous 12 months.

##### Retention

ALHIV active in care basing on most recent scheduled clinic appointment which should be at or less than 28 days, using the United States’ President’s emergency plan for AIDS Relief (PEPFAR) [18]. We measured retention among the ALHIV at one year, categorizing outcomes as active (if ALHIV was within their most recent appointment), missed appointment (no clinic encounter on scheduled appointment but within 28 days), dead (those confirmed to have died), lost to follow-up (untraceable individuals after at least four attempts) and transfer out for those who shifted to other facilities.

### Data management

#### Data collection

##### For quantitative data:

We used a questionnaire to abstract secondary data from patient files, registers and Uganda electronic medical records, entered into an online database hosted on KoBo toolbox [19]. The tool was tested in TASO Gulu and TASO Masindi centers of excellence, making necessary adjustments before final deployment. Teams of three individuals, including YAPS, counsellors and Monitoring and Evaluation officers were trained and used to conduct data abstraction. Data was downloaded and exported to Microsoft Excel, version 2019 for basic cleaning and preparation for final analysis in STATA Corp, version 15.0.

##### Qualitative data:

We trained a team of experienced research assistants from Makerere University School of public health for one day, to collect qualitative data using interview guides. Key informant interviews (KIIs), in-depth interviews (IDIs) and focus group discussions (FGDs) were used to gather data from respondents. Health workers including clinicians, counsellors, medical doctor, nurses and the YAPS who worked closely with the ALHIV offered their expert perspectives through KIIs, while caregivers and ALHIV responded to the IDIs and FGDs. For FGDs, groups of 6 ALHIV with non-suppressed VL, six ALHIV with suppressed VL, six caregivers of ALHIV with suppressed VL and six caregivers of non-suppressed VL. Each FGD took an average of 45 mins, IDIs 1 hour and KIIs similarly took 1 hour. In total, we conducted 35 interviews. All interviews were conducted face-to-face within the clinic setting, in comfortable rooms without the presence of non-participants to ensure confidentiality. Interviewers used semi-structured interview guides with translations from English to Luganda, Ateso and Lumasaba languages. Data was collected as audio-tape recording and complemented with field notes. Respondents were informed of field notes being taken. Finally, interviews stopped when the team could no longer identify any new themes.

#### Data Analysis

##### Quantitative Data

Analysis was completed in STATA Corp, version 15.0. Univariates were summarized as frequency, and percentages. Pearson’s chi-square was used to determine association among the various categorical variables, at a confidence interval of 95%. Variables with P-value less than 0.2 were considered for multivariate analysis. To determine factors associated with retention and VLS, logistic regression was used, with odds ratios (OR) preferred for reporting magnitude of association using 95% confidence interval, at P < 0.05.

For measurement purposes, factors were categorized into individual (adherence, age, sex, age at diagnosis, school going status, TB status, disclosure status); facility-level (provision of DSDM, provision of OVC services, treatment optimization) and community-level (distance to facility, caregiver relationship, caregiver HIV status and caregiver VL suppression status)

##### Qualitative Component

Audio recorded data was transcribed into texts, translated to English and entered into Atlas ti. for analysis. Four research assistants conducted the initial coding, working with BO, AIO, AK and DK, developed codebooks that were used to complete the thematic analysis. The four research team members then met virtually to refine the codes to ensure they made sense. Major themes and sub-themes were developed in line with the study objective. Key statements were transcribed verbatim and reported as appropriate.

##### Ethical Consideration

## Results

A total of 59 respondents participated in the study, majority of whom being peasants, those with post-primary education, and aged at least thirty years.

From Table 2, a total of 533 records were considered for the analysis. The mean age was 15 years (SD=) and majority of the ALHIV were females (54.2% vs 45.8%). Importantly, all the adolescents were on optimal ART regimens, predominantly, DTG-anchored drugs in line with national efforts to ensure all PLHIV receive optimal therapies. Further, we note that up to 38% of the ALHIV were living with non-biological parents which could have resulted from the demise of both parents. Indeed, a recent UNICEF report indicate that 13.9 million children aged below 18 years, world-wide had lost one or both parents to AIDS-related illnesses over time [2]. This could affect social support systems, resulting in poor treatment outcomes for the ALHIV. Importantly, majority of the adolescents were in school (69% vs 31%). School is protective and improves one’s abilities to discern life’s challenges and opportunities quite well.

In regard to viral load, we found that all the adolescents had at least a VL test done within the previous 12 months with an average VLS rate of 74.9%. This is way below the expected 95% and barriers were explored in the qualitative component, presented later in the manuscript. For the 134 with non-suppression, 34.1% had low level viremia while the remaining 65.9% had high level viremia. These will be enrolled into the study for enhanced support, enshrined in the OTZ model. As for retention at one year, it was impressively high at 95.9%, above the expected target of 95%. The attributions will be further highlighted under the qualitative findings . However, quantitatively, we attribute this good retention rate to the implementation of differentiated service delivery including multi-month dispensing and community-based ART delivery approaches. In addition, the high rate of HIV status disclosure (97.9%) could also have played a role as well as treatment optimization and the OVC platforms. Further, adherence was generally good, at 92%. However, it is important to remember the subjective nature of the measurement used on this occasion. The measurement was based on self-reports by the clients and adherence levels categorized as poor, for below 84%, fair for 85–94% and good if more than 95% as per national standards. This approach could lead to an over-estimation of optimal adherence levels.

From Table 3, several factors were associated with retention and VLS. For retention, disclosure of HIV status (P = 0.018), distance to facility (P = 0.042), multi-month dispensing (P < 0.001), OVC status (P < 0.001), caregiver relationship (P < 0.001), school going status (P = 0.023) and age at diagnosis (P < 0.001) were all significant factors. On the other hand, multi-month dispensing (P < 0.001), current DSDM (P < 0.001), current ART line (P = 0.002), current regimen (P = 0.031), and adherence (P < 0.001) were all associated with VLS. Conversely, caregiver HIV status, TB status and current DSDM were not associated with retention. Similarly, HIV status disclosure, school going status, TB status, caregiver HIV status, age at diagnosis and nutrition status were all unassociated with VLS.

In our analysis in Table 4, certain key predictors emerged as significant determinants of retention on Antiretroviral Therapy (ART) at the 12-month mark. Notably, individuals with higher current weight were found to be significantly more likely to be retained on ART, with an adjusted odds ratio of 1.1060 (P = 0.045, 95% CI: [1.0206, 1.1985]). This implies that for each unit increase in weight, the odds of retention increased by approximately 10.6%.

Moreover, the influence of school attendance on ART retention was profound. Students currently attending school exhibited a considerably higher likelihood of retention (adjusted OR: 0.1488, P = 0.041, 95% CI: 0.0240, 0.9218). This indicates a nearly 85.12% reduction in the odds of non-retention among individuals attending school.

Caregiver dynamics also played a crucial role in predicting retention. In the unadjusted model, individuals under the care of a guardian were significantly less likely to be retained on ART (OR: 0.2738, P = 0.01, 95% CI: 0.1095, 0.6844). While the significance dimifinished slightly in the adjusted model (adjusted OR: 0.4861, P = 0.463, 95% CI: 0.0709, 3.3353).

Another critical factor influencing retention was the caregiver’s viral load status. Non-suppressed caregiver viral load significantly decreased the likelihood of retention on ART in the unadjusted model (OR: 0.0478, p = 0.002, 95% CI: 0.0069, 0.3369). This implies that individuals under the care of caregivers with non-suppressed viral loads were only 4.78% as likely to be retained on ART compared to those with suppressed viral loads.

Additionally, the disclosure of HIV status played a notable role. Individuals who had not been disclosed to, their HIV status were significantly less likely to be retained on ART in the unadjusted model (OR: 0.1793, p = 0.04, 95% CI: 0.0363, 0.8858). This fifinding retained significance in the adjusted model (adjusted OR: 0.3610, p = 0.386, 95% CI: 0.0361, 3.6078), suggesting a substantial impact on retention outcomes.

These findings suggest a multifaceted nature of factors influencing ART retention. While current weight, school attendance, caregiver relationships, caregiver viral load, and disclosure status were identified as significant predictors, addressing these factors collectively may offer a more comprehensive approach to improving retention rates in individuals on ART

### VIRAL LOAD SUPPRESSION

From the analysis in table five, it is clear that adherence scores played a crucial role, with both fair and poor adherence significantly decreasing the odds of viral load suppression. In the adjusted model, individuals with fair adherence had a reduced likelihood of suppression (p = 0.002, OR: 0.066, 95% CI: 0.0115, 0.3850), and a similar trend was observed for those with poor adherence. This suggests that maintaining good adherence on ART is essential in achieving viral load suppression.

Current weight emerged as another significant predictor, indicating that for each unit increase in weight, the odds of viral load suppression increased by approximately 3.2% in the unadjusted model (p = 0.03, OR: 1.032, 95% CI: 0.9813, 1.0843). However, this significance was not maintained in the adjusted model, emphasizing the importance of considering other factors.

Interestingly, the type of ART line showed significance in both unadjusted and adjusted models. Individuals on the second line had significantly reduced odds of viral load suppression (p = 0.001, OR: 0.242, 95% CI: 0.0873, 0.6724), indicating that being on the second line of treatment is associated with a lower likelihood of achieving viral load suppression. The significance of this association was not observed for those on the third line.

The current Differentiated Service Delivery Model (DSDM) approach also played a role in viral load suppression. Notably, individuals under the FBIM (Facility-Based Individual Model) approach had significantly higher odds of suppression compared to their counterparts in Facility Based Groups (p < 0.001, aOR: 0.152, 95% CI: 0.0226, 1.0180). In contrast, those under CDDP (Community Drug Dispensing Point) had lower odds of suppression (p = 0.001, aOR: 2.341, 95% CI: 0.5227, 10.4851). This suggests that the choice of DSDM approach may influence the likelihood of viral load suppression.

It should be noted that duration on ART, current age, school-going status, caregiver relationship, caregiver current VL status, OVC status, Multi Month Dispensing (MMD), and distance to the facility did not show significant associations with viral load suppression in either the unadjusted or adjusted models.

### Qualitative findings

We classified barriers and facilitators into three main themes: individual level, facility-level and community-level factors. Key barriers identified thus, include:

#### Individual level barriers.

Internal stigma, was a commonly cited barrier to both retention and viral load suppression. ALHIV feel uncomfortable in environments where they have not disclosed their HIV status, and eventually abstain from swallowing drugs, culminating in viral non-suppression. This includes schools for the school-going ALHIV, unfamiliar hospital settings and those in sexual relationships where the spouse is ignorant of the adolescent’s HIV status. In those circumstances, the adolescents harbor a feeling of being identified by onlookers, to be living with HIV, with perceived negative consequences, sometimes due to previous experiences. Consequently, the ALHIV tend to conceal their HIV status which sometimes results in poor adherence.

“The challenges that I always experience from taking the drugs sometimes like now when I am at school and ever since my childhood, I have been in boarding you know with boarding life it is not easy sometimes you fear swallowing the drugs when you see the people there like when I was in primary, I used to be shy…”

female non-suppressed adolescent

Another adolescent said,

“First of all, we like bragging a lot, for example when you are dating a girl and when she comes home, for example she has finished a full week, you will not show her that you are on treatment, you will not swallow the drugs.”-male non-suppressed adolescent. This was further cemented as follows, “it happens mainly with adolescents like us may be when you engage yourselves in relationships. I am talking this out of experience you engage yourself in a relationship yet your boyfriend doesn’t know that you take drugs so it happens that you went out with him and the time has reached for you to take your drugs but because you don’t want him to know you just let the day go like that”

- female suppressed adolescent.

The adolescents need to be supported to overcome their own internal stigma and helped to disclose HIV status to significant others. Another barrier highlighted was poor adherence. As already demonstrated quantitatively in this study, good adherence was found to be associated with VLS, (P < 0.001).

“I realized, she just picks from the container, then she used to go and hide them under her bed, then you ask her have you swallowed? then she says yes”

- caregiver of a non-Suppressed ALHIV.

This was a common experience reported by caregivers and even health workers. It thus calls for empowerment of the adolescents to appreciate the need to adhere well and also treatment support from caregivers or peers. In addition, it is worth noting that some adolescents become non-adherent merely to explore its potential effect on their health as one health worker observed:

“as they come here they will discuss that do you know for me I have taken now one week they told me to come on such a day I did not go I have come but I am okay, next time you are doing viral load the very child is suppressed and then they will say you see they tell us if you miss your drugs you will get non-suppressed but for me I have not so, they try some of these things some of them intentionally refuse to come for appointments because they want to first stop taking drugs and see what will happen actually”

-counsellor, TASO Mbale CoE

Sometimes, this is due to negative peer influence. For example, in one session, it was reported that ALHIV can by consensus among themselves, agree to abandon ART for some time as noted in the quotation below

“Now mine swallows the medicine very well but sometimes when they are in a group and they sit with their fellow positive friends so they say let us just leave this because now we are grown up and they miss like one day and then the following morning”

-caregiver for a suppressed adolescent.

Akin to the challenge of poor adherence is the issue of drug or treatment fatigue. Considering that 43.5% of the ALHIV were diagnosed by the age of two years and overall, 95% by 10 years, means that majority have been on ART for a long time. Thus, some of the ALHIV may become fatigued from having to swallow drugs daily. Here, one adolescent notes;

“Swallowing this drug everyday you can get tired, you dodge, you go and keep the medicine, they ask “have you swallowed? you say yes, I have swallowed, yet you have kept. Because now, like today you have swallowed, tomorrow again and the next day the same medicine, you keep asking yourself am now tired”

-non-suppressed male adolescent.

It is thus, vital for health workers including peers to empower the adolescents in order to have a positive outlook to life. Also, when it becomes available, ALHIV could be prioritized for long-acting injectable ART as one respondent requested:

“There was an idea health workers had told us that it was suggested and that is giving us injections for ARVs instead of tablets because if a child is given an injection, you can even send him to stay with the grandmother and you also do something until the next appointment date which is not possible with tablets which have to be swallowed daily and at times there are people you don’t want to know that you are taking ARVs because it can affect you or the child, but if it is an injection, no one can know that you are receiving ARVs.”

-caregiver, suppressed adolescent-TASO Mbale

Difficulty in finding transport money was another important barrier identified. Notably, 61% of the adolescents live more than 5 kilometers away from their respective health facility. This, thus requires that adolescents find some means of transport such as “boda-boda”, non-motorized bikes or taxis in order to access the health units. However, this requires money and yet for some adolescents, especially those who live with grandparents or without any caregiver at all, find real Difficulty in accessing such resources. One adolescent noted as follows,

“Yeah, a lot of challenges, you see am from Ngora the time the day reaches, you don’t have even transport, the grandmother may have to look for transport, it may take 2–3 days that’s when you have to come, yeah sometimes I miss because of transport.”

-non-suppressed female adolescent.

Scaling up community-based drug delivery points could alleviate this challenge as it has potential of reducing the distance that individuals have to cover in order to access services. In addition, skilling the adolescents could also empower them to produce marketable goods or services that could attract resources and enhance their economic prowess as suggested by one respondent;

“Maybe financially, if there can be some chance of getting some funds because sometimes things can be hard, a day comes when you don’t have money and may be if we can have some sort of IGA (income-generating activities) so that we can get his transport so that when the day comes, he does not miss to pick his drugs.”

-caregiver, non-suppressed adolescent

#### Facility-level barriers

At facility level, we identified also some important barriers. Firstly, lack of social activities within the facilities. It is worth noting that the opportunities for the adolescents to meet and interact periodically is important for creating a strong social fabric that glues together peers. This bond normally goes a long way in enhancing individual’s self-esteem, treatment literacy and overall positive outlook to life. Unfortunately, the adolescents observed that these were missing, denying them the great opportunities to meet, share experiences, learn from each other and encourage one another in order to improve well-being and health outcomes.

“I think some of our colleagues why they are not suppressing is because there is no motivation that used to exist, like food, the things that they used to give, the games that were here now when you come here you stay hungry the whole day so, someone might think even if I go there I will stay hungry I am not going to collect medicine sometimes when you swallow medicine it can also give you a problem it needs you to first eat so, such things can make someone to say for me I don’t have what to eat so someone can stop taking medicine and the viral load will still remain up.”

Suppressed adolescent, FGD.

It is also important to note that the COEs used to provide lunch for the adolescents during clinic days as well as social activities including various games which has since stopped due to high prioritization of funds. As some adolescents suggested, a rejuvenation of the activities would re-ignite their passion for living and attending clinic appointments as it did before. To this, one adolescent said:

According to me I would like TASO to provide us with things like games and at least let them provide us with like when we meet like every month the adolescents have a clinic. Yes, in one particular day in a month adolescents meet, so whenever you meet, you interact with others at least as you are waiting for your medicine you should be playing something may be computer there, games chart with others, food, you come here maybe you came at 8:00 am and you may end up going back at 2:00 pm or 4:00 pm that period of time is really affecting.

-FGD, Suppressed adolescent.

Prolonged waiting time was another important barrier observed. PEPFAR [20] recommends that PLHIV need to take less than one hour accessing services in a facility to motivate clinic attendance and stimulate retention in care. However, it was noted that sometimes the clinics are heavy, leading to prolonged waiting time. This potentially demotivated some ALHIV from attending scheduled clinic appointments as one adolescent observed as follows:

“Sometimes people are many, that when you reach here as in the line like at 11am there, you may leave here at around 4pm, by the time you reach where I stay, like for me am from Ngora, it will be at around 8pm there.”

Female non-suppressed adolescent.

Perhaps, clinics could consider scaling up Differentiated Service Delivery Model (DSDM) especially the community-based approaches and also Multi-Month Dispensing of drugs (MMD) as well as better appointment system to spread out evenly, the number of clients per clinic day.

Finally, some respondents also noted drug stock-out as another barrier. This was mainly cited by those on third-line. It is important to remember that thirteen adolescents were on third line ART, yet its stock remains unreliable country-wide. To this, only one warehouse (national medical stores) procures and supplies third-line drugs across the country limiting its access sometimes. In addition, prolonged turnaround time of HIV drug resistance test results was noted as another impediment, with clinicians having to wait for as long as six months sometimes. This delays appropriate clinical decision-making which in turn condemns the affected ALHIV on failing regimens for unnecessarily long. Currently, only the central public health laboratory and Joint Clinical Research Center conduct HIV drug resistance testing in Uganda, partially explaining the prolonged turn-around time. The respective quotations are presented as follows,

“From here, maybe the drugs like we love drugs like for people who are in the third line so I am on third line and sometimes you come and then they tell you the drugs are not there and then you go back home without the drugs”

-suppressed adolescent.

“Then another barrier is the turnaround time for example maybe okay at least some people with the viral load the time has shortened a bit but our major problem here is the HIV drug resistance it can literally take like six months when the results are not yet out then even we were advised that even after the six months if that HIV drug resistance results have not come back we take another sample so it means it is going to take us a year to reach the decision for this particular child so you find that this one is writing away drug resistance results and now you can’t proceed to another arrangement because you want to wait for that file to come and then make a decision so it takes about six months to a year”

-clinician, TASO Mbale

Uganda Ministry of health could consider mechanisms such as decentralization of HIV drug resistance testing and delivery of third line drugs to address this gap.

#### Community-based barriers

The study also identified important community-based barriers to retention and VLS. One commonly cited barrier was external stigma and discrimination. The respondents noted this, occurring from the wider community but also in schools, as demonstrated in the quote below:

“My mother disclosed to that teacher, that teacher had no secret he went on telling, people, telling people. Children did not want to sit with me on the desk, then it reached time when I hated myself and I told my mother to get for me another school, what she did.”

-male non-suppressed adolescent

Stigma and discrimination are selfish vices that deprive victims of the opportunity to peacefully live and exploit their full potential. It can lead to reduced self-esteem, a feeling of self-unworthiness and full-scale mental ill-health if unaddressed. Suffice to mention that ALHIV are often more adversely affected by this experience given their stage of growth that is often characterized by many physiological, neurocognitive and physical changes. There is thus, need to continuously sensitize the communities including teachers to elevate awareness to this vice that can lead to catastrophic outcomes. To this, one adolescent mentioned,

“They used to tell me that, “you any time you are dying, people from Bududda are going to buy all your land, any time you are dying “so for me I did not report to any one whenever they say so, I just keep quiet”

-male non-suppressed adolescent.

It can also stimulate adverse behavior including rejecting drugs among the adolescents who are affected, as noted in the quote below;

“Mine decided to throw the drugs away, you hear that? because the colleagues were laughing at him, he didn’t know why he was taking the drug, so when she saw the drug, the colleague said “eeeh, this drug we saw our grandmother also used to take the same drug, so you are taking drugs for HIV, then he became shy, then he throw the drugs in the dust bin. “

-caregiver, non-suppressed adolescent.

Further, respondents revealed lack of food as a credible barrier to retention and VLS. As one respondent observed,

“Some of us life is very difficult even to get what to eat sometimes it’s very difficult. you know staying with grandparents, they only think that digging is the only important thing in their life, so when they don’t uproot something for sell, you will not get money and even single coin in your life, even food. at times other seasons rains delays so, even cassava may not be there at home, …you just go to other homes”

- female non-suppressed adolescent.

The lack of food frustrates optimal adherence, leading to non-viral suppression. Food and nutrition generally are important in improving absorption and also tolerability of drugs, potentially, optimizing overall bioavailability of antiretroviral drugs. Enhancing the capacity of adolescents, especially the ones outside school and their caregivers, to actively participate in food production could alleviate this burden of food shortage. This could be done through collaborating with selected community-based organizations, already involved in such projects to skill the ALHIV and also routine food/recipe demonstrations within the facilities. One adolescent stated,

“You know the good thing with food, those days when food was provided like some of us who come from far, so when you eat food, you find that you can be able to weight for your medicine when you are satisfied and it helps to suppress and you will be like when I go there, I can eat something so, that thing encourages. So, even the child will be like I want to suppress so that next time when I go back there, I will get food so there is that motivation.”

FGD, suppressed adolescents

On the other hand, inadequate social support was yet another major barrier cited. Social support is critical in chronic care and without it, the disease condition can overwhelm the system.

The biological father doesn’t want to see the child and does not want to know that he has a child, he said that “those are HIV affected children I don’t want them, let them die so that I can get condolence and I eat “and even when the child goes to the father, he doesn’t give him anything even a single coin.” Caregiver of a non-suppressed adolescent.

This inadequate social/family support expands beyond the economic facilitation to other dimensions such as proper supervision of ALHIV to ensure they are indeed adhering well. Some caregivers assume that the adolescents are old enough to manage their own health, thus neglecting the important role of providing treatment support as one caregiver highlighted below:

“Some parents why their children are non-suppressed is because they tell them that go and swallow drugs, the child will go get drugs and discard, and for you, you will think he/she has swallowed and when you go to the clinic and they check the viral load, will be high. the problem will be that you don’t give and observe the child when he/she is taking the drugs.”

Caregiver of a suppressed adolescent.

In this study, some eight (8) ALHIV were without caregivers, exacerbating this barrier even further. Moreover, some of the ALHIV had unstable caregivers, moving from one to another. This inconsistency affects optimal social and economic support.

“I would also look at involving the parents into this when we are trying to make a decision or we are trying to look into this walk together as they are giving the medication the parent should be fully involved in what is happening to these children. The experience I have had with home visits, there are homes you visit the child now today you have found the child is in this home and the next time you go to do a home visit the child is no longer staying there they are now staying with a sister somewhere else now maybe when you go to the other home where she stays with the sister you find that this child is not there”

-clinician, TASO Mbale

This instability deprives the adolescents of good nurturing as most times, nobody is there to take full responsibility. It also frustrates the efforts of health workers who attempt to provide treatment literacy and empower the caregivers due to the frequent changes in caregivers. Nonetheless, some caregivers just forget their responsibilities over time and assume that their work stops at picking up drugs for the adolescents. To this, health-workers need to periodically engage the caregivers including teachers to continuously sensitize them on their basic responsibilities, as suggested by some respondents indicated in the quotes below:

The counsellors should regularly invite parents or caregivers of those adolescents for counselling sessions on how to support these adolescents because when caregivers take time without having such sessions, they tend to forget everything and relax. So there should be continuous sessions for caregivers. adolescent should be invited for the sessions such that he/she also knows what to do when it comes to adherence and how to live with their peers at school

-caregiver, suppressed adolescent.

This was further stressed by a counsellor:

“But also talk to the caregivers to play their roles towards adherence. To help the adolescents to check on their appointment dates, 2-to help the adolescents and children to reach the clinic, 3- observe a treatment process at home, we talk of directly observed therapy, and then 4- provide basic needs like food and clothes such that a child does not feel like not taking drugs.”

Counsellor, TASO Soroti

In terms of facilitators to both retention and VLS, we also categorized them as individual, facility and community-levels.

#### Individual level facilitators

Knowing one’s HIV status was a good motivation for optimal retention and VLS. As noted earlier, some 11 ALHIV had not yet been disclosed to and yet disclosure was associated with good retention in care. Indeed, those who were disclosed to their HIV status were seventy times more likely to be retained in care (AOR = 0.014; CI, 0.0468–0.2247; P = 0.03). Coupled with older adolescents disclosing their HIV status to others, this can attract better social support with good retention in care and VLS as highlighted in the quotation below:

*“He advices me to take my medicine at the right time and before taking drugs you must first eat something I always take in the morning and he tells me that make sure that there is tea in the morning for to take before you swallow your drugs*. Yes, I told him that I am positive so we use condoms and he told me that if you stop taking medicine, I will also leave you.”

Female virally suppressed adolescent.

“Maybe to talk to the adolescents the reason why they are taking their drugs, how to do health education where we have experience sharing like you found us doing some cession there where by some of them at first, they were shy others were like oh I thought I was alone so it brings them hope.”

−a YAPS, TASO Mbale

This was stressed further as follows

“if you disclose to them before them finding out on their own; once they can understand then you disclose to them, they will know the reason as to why they are taking the drugs and also know why they are alive up to that time, and that is one of the things that can help us; disclosure to them and also to significant family members because you”

-medical doctor-TASO Soroti

Without doubt, disclosing HIV status is beneficial in empowering the adolescents to take more central role in their own health.

Good adherence is another important facilitator. As already alluded to in the previous section, good adherence is associated with VLS (P < 0.001). Respondents observed that those who swallow their drugs properly had suppressed their viral loads as well. Considering that all the adolescents in this study were on optimal regimens (DTG-based or protease inhibitor-anchored), means that with good adherence, the ALHIV should ideally suppress their viral load. One caregiver observed the effect of good adherence in the following quote:

“He swallows the medicine very well because we came here and they told him the time that he should take the medicine and when that time reaches, we have to tell him or even when he doesn’t remember we try to remind him and tell him to swallow the medicine but he has never missed ever since he started swallowing medicine.

-caregiver of a suppressed adolescent

#### Facility level factors

Respondents identified provision of adolescent friendly services as a facilitator. The facilities provided differentiated services including community ART delivery approaches, multi-month dispensing, appointment reminders, presence of the YAPS, clinical and psychosocial services. These services inspire adolescents and their caregivers to adhere to their scheduled appointments. Moreover, the ALHIV also noted the good attitude of health-workers in the two COEs as magnified in the quotations below

“I have found it good in that when we reach the health workers attend to us very well, they don’t ask for many things, they ask if the patient is swallowing the medicine very well and we tell them that yes the medicine was well swallowed so that is the good thing here. Another thing why the clinic is okay is that I might forget of my appointment they call me and remind me of the appointment and immediately I also say it is ne I am coming and I organize myself and come so they remind me”

-caregiver of a suppressed adolescent.

Another respondent stated that:

*“Sometimes I fear to disclose to someone who is not of my age everything but I can disclose to someone of my age everything.”- virally suppressed adolescent*. This statement underscores the importance of implementing the YAPS program to enhance quality of HIV services among the ALHIV. This was further magnified by a YAPS who noted that adolescents were more comfortable *interacting with younger health workers as opposed to the older people due to the massive age gap.

“they want a young person may be who is a doctor to attend to them someone who understands them better because you may find that may be some one of 60 years or 40, or 50 years so at times they don’t fill comfortable sharing issues with them and you find someone has a problem and they come to see the doctor and goes back with it so when you interact with them so why didn’t you see the doctor that means there is fear to share with those elderly people so they want their age range.”

- YAPS, TASO Mbale

The downside though is that whenever the ALHIV fail to find familiar health-workers, some of whom feel frustrated and fail to seek the necessary service as noted by a clinician in the following quotation

“One of them is that most of them are accustomed to a particular clinician and also the YAPs. In the event that they come to the centre and those ones they want are not there, they are also out of place and that alone affects their interaction with you. Sometimes some of them may have complains and challenges that ideally, they are supposed to air out and you support them but because they are used to particular individuals who may not be there at that time during that particular clinic day, they don’t really bring out and that affects your interaction.”

- Medical doctor-TASO Soroti

#### Community-level facilitators

Social support. As already noted, social support is fundamental in achieving good health, especially with chronic care. Indeed, respondents observed socio-economic provisions as key in motivating good retention and VLS among the ALHIV. This support is expansive, transcefinding through family/household to wider community-level. It includes reminding the adolescents of their scheduled clinic visits, caregiver supervision of adherence and provision of transport money for clinic visits as well as decent food. We illustrate these with the following quotations

“On the side of nutrition much as the situation is not so good, but at least we endeavour to see that after taking his drugs he has to have something to take like porridge or tea even if we don’t have escort and also lunch, he has to eat in time as well as supper.”

-caregiver of a non-suppressed adolescent

Further elucidation from one adolescent who reported on the support they received from a parent that enables them to remain suppressed.

“For me whenever I get medicine, I take it home but it is my father who gives me to take because sometimes I forget”

-suppressed adolescent

And in schools: *“Yes, like at school the administration they know then the matron because it the matron who used to even give me drugs and she is the one who used to keep my drugs.” -suppressed adolescent*.

It is thus important for health workers to empower the adolescents and their caregivers to disclose HIV status of those in schools to the administrators. This can foster support from an informed point by the school infrastructure, limiting adverse experiences such as stigma and discrimination that is observed sometimes in the institutions of learning.

## Discussion

Overall, in this study, retention rate at 12-months was high at 95.9%, surpassing the 95% expected target. To the contrary, VLS rates among the ALHIV in the setting was sub-optimal, at 74.9%, far below the expected 95%. To this, we identified several barriers and facilitators, categorized as individual, facility- and community-level factors which HIV programs could consider in attempting to improve VLS in the sub-population. The findings will also act as a baseline for the adaptation and implementation of OTZ to the TASO setting.

The good retention rate is much better than reported in most previous studies. For example, Muwanguzi et al., reported 65% among the adolescents and young people aged 15–24 years [5], 29% by Izudi et al., [21], 69.5% by Cluver et al., [7] and by Zanooni et al., 89% [22] and 35.7%, Nimwesigwa et al., [11]. As for VLS, it was higher than reported by Hlophe et al., [4] at 55%, 65% by Simms et al., [9], 62% in Kenya [23] but lower than by Tugume et al., [6] at 81%. However, it is important to note that the latter was based on a cut off of 1000 copies/mL rather than the 200 copies/mL used in this study. Nevertheless, the good treatment outcomes are attributable to a myriad of factors that include provision of adolescent health friendly services as enshrined in the WHO guidelines [24]. These include differentiated service delivery such as community drug delivery approaches, good attitude of health workers toward the ALHIV, proper appointment management systems, multi-month dispensing of ART and the presence of YAPS.

These factors have indeed been documented elsewhere as contributors to optimal retention in care and VLS among the ALHIV. Peer driven models such as CATS and Teen Clubs have demonstrated positive impact on improving retention among the adolescents in Zimbabwe [16, 25]. The YAPS model is designed to enhance peer-to-peer support including adherence counselling, building on socio-cognitive theory which contends that individuals are more likely to be influenced by their peers [26]. Indeed, this finding underscores the invaluable contribution of the YAPS program in improving treatment experience of the ALHIV. Uganda started its implementation in 2019 to enhance peer-driven quality of HIV services using age-appropriate messages and techniques [11]. As already noted, ALHIV are more comfortable, opening up to someone of their age than much older people and thus the peer-driven approach is an important catalyst to a person-centered HIV care. Further, other differentiated service delivery models (DSDMs) such as the Zvandiri intervention posted 98% retention rate at one year[27]. DSDM, a person-centered approach, remains a cornerstone in revolutionizing HIV services, particularly among the adolescents [28] as it focuses on delivering services as per individual client needs. Interestingly, these factors were associated with low retention in one study conducted in western Uganda [11]. The study found that ALHIV in facilities implementing the YAPS model and enrolled into the differentiated service model (facility-based groups) were more likely to be lost to follow-up. This likely reflects the complexity or uniqueness of ALHIV needs and implementation fidelity (or lack there-of) of the interventions, in the different settings. In this study, the ALHIV in facility-based groups were more likely to be virally suppressed compared to those in community-based models. Further, the adolescents who received ART refills for at least three months were more likely to be retained in care than their other counterparts [AOR 95%.CI: 32.6287 (5.1446, 206.9404) P < 0.001]. Both community-based DSDM and MMD counteract the challenge of lack of transport money. Without doubt, adolescent friendly services are critical in improving retention. As Ritchwood et al., noted, health-worker-client relationship plays a central role in optimizing retention among the ALHIV [29]. In their study, Ritchood observed that the ALHIV referred to their service providers as ‘family’ and facilities as home. It is therefore unsurprising that friendly health workers were identified in this study as motivators for adolescents and their caregivers to remain in care, a similar finding by Cluver et al., in a South African study [7]. Thus, these interventions need to be up-held in-order to sustain the good retention rate. However, community-based ALHIV need to be supported so as to attain the required level of VLS.

As part of person-centered service provision, health systems need to focus on the older adolescents, aged above 14 years, since they were more likely to be virally suppressed compared to those aged 14 and below [AOR:95%CI 0.342 (0.1180, 0.9885) P = 0.048]. The adolescents in this age category experience the most profound psychological, physiological and physical changes [30] hence need to address their specific needs. This is worse for those who are out of school. It was also found that school going ALHIV were more likely to be retained in care than those out of school [AOR:95% CI 0.1488(0.024, 0.9218) P = 0.041]. School is protective against HIV [31] and possibly other disease conditions. It is also likely that those in school are more enlightened about their health and hence become more proactive compared to their counterparts. HIV programs need to tailor interventions that reduce the gaps among the out of school ALHIV and especially those age above 14 years to reduce the inequity. These could include skilling programs that empower them to enable the ALHIV to access resources and platforms that strengthen their capacity.

In addition, disclosing HIV status to the ALHIV was also an important enabler. Disclosing HIV status to the ALHIV and the client themselves to others can be a complex task, considering the potential repercussions such as stigma, fueling family conflicts and hazardous behavior [32]. It is thus a process, often involving health professionals and caregivers, working in concert to reveal to children and adolescents that are perinatally infected about their HIV status. In Uganda, full status disclosure is expected by age of 12 years [33]. Disclosure empowers the adolescents to improve adherence and embrace positive living which results in improved retention and VLS. On the other hand, disclosing HIV status of ALHIV to others such as sexual partners or school administrators can ignite a positive feedback loop. It can encourage provision of necessary support that the ALHIV require including psychosocial and financial from those disclosed to. In addition, ALHIV often find difficulties in leaving schools to attend clinic visits, in case of non-disclosure, a similar experience reported by Kimera et al.,[34]. Thus, health systems need to prioritize offering support for disclosing HIV status in order to rip its full benefits. Suffice to note that Uganda’s ministry of health current guideline (2016 version) on disclosure is fairly insufficient and urgently requires revision to offer additional support to the health workers. For example, the timelines for initiating and concluding partial disclosure remains vague, unlike in other countries [33].

Further, effective social support system is another important enabler. In their study, Lypen et al., [35] identified four different types of social support; emotional, informational, appraisal and instrumental. They also demonstrated a wide range of sources of social support which such as family members, friends including spouses, health workers, teachers and religious leaders. Social support provides the necessary oil for lubricating the engine that runs the chronic care machine. As Damulira et al., [36] reported in their study, caregiver/family social support was associated with self-reported adherence among the ALHIV in Uganda. Indeed, Okonji et al [37] report that family-centered interventions were critical in improving adherence and retention among the Adolescents and young people living with HIV in their study. Thus, strong cohesive families remain the bed-rock of achieving optimal treatment outcomes among the ALHIV and programs need to prioritize family centered approach in line with the Uganda national focus of integrated community service delivery model (ICSDM) [13]. As part of ICSDM, other community service organizations/community-based organizations (CSOs/CBOs) could be engaged to offer additional support to the adolescents and their caregivers, including facilitating skilling programs or income generating activities. Our finding indicates that the ALHIV who were enrolled onto the Orphaned and Vulnerable Children (OVC) platform, an activity run by a CSO, were more likely to be retained in care than their other counterparts [AOR:CI 0.262(0.0831, 0.83) P = 0.023]. This finding validates the need for multiple stakeholders in managing the HIV response among the ALHIV.

Lastly, good adherence was an important predictor of VLS in this study. Those who reported fair adherence were less likely to suppress their viral load compared to their counterparts who reported good adherence scores [AOR:95% CI 0.066 (0.0115, 0.3850) P = 0.002]. Adherence, is a precursor to HIV prevention, optimal retention and VLS [38]. Poor adherence, characterized by missing pills, can lead to development of drug resistance, transmission of HIV infections and poor treatment outcomes. Several respondents in this study highlighted factors that affect adherence, including availability of food and good feeding, presence of responsible caregivers who supervise ART adherence of their ALHIV and presence of drugs. It is perhaps not surprising that caregivers whose viral loads were unsuppressed were also likely to take care of adolescents with non-suppressed VL [AOR:95%CI 0.144 (0.0232, 0.8944) P = 0.038]. This was similar to the findings from a Kenyan study [23]. Moreover, we also found that ALHIV on second line ART were less likely to suppress their VL. This could portray failure of HIV programs to identify and address the underlying adherence barriers before switching to second line, including the issue of treatment fatigue. Efforts need to be geared toward addressing adherence barriers and ensuring that ALHIV remain adherent to medication, in order to achieve and sustain VLS.

We recognize important study strengths and include: The mixed method design ensured robustness of undertaking, with qualitative and quantitative approaches complementing each other. This improved the overall credibility of our findings. Secondly, the design and deliberate approach employed to collect and analyze qualitative data provided rigor and ensured reliable findings which inspire confidence in interpretation and utilization. Finally, the use of routine programmatic data for the quantitative component provided credible and reliable information since it likely reflects the true situation on the ground.

Further, we also acknowledge some key weaknesses of this study: Firstly, it only considered two TASO sites without involving any public health site. This can potentially diminish its wide applicability in other settings, given the obvious differences in the capacities of TASO sites and public health facilities. Nonetheless, the findings can still be useful in a wide range of settings as adolescent challenges may cut across. Secondly, the use of secondary data for the quantitative analysis led to exclusion of some ALHIV due to incompleteness of crucial information. The missed individuals could have added value to the findings. Nonetheless, the use of a census approach ensured that the sample size was large enough to counteract the potential effect of exclusions. Thus, the findings are still credible.

## Conclusions

1. Our findings indicate that short-term retention among the adolescents living with HIV in TASO Soroti and Mbale COE was high, at 95.9%. It is thus possible to use limited resources and achieve optimal retention rates among the ALHIV through offering adolescent health friendly services such as multi-month dispensation of ART, community drug delivery approaches and disclosing HIV status. This finding is testament that PEPFAR’s and Uganda’s heavy investments in HIV programming have not been in vain.
2. Despite several efforts and interventions, VLS among the ALHIV remained sub-optimal, at 74.9%, far below the expected 95%. Moreover, the findings portray insufficient involvement and engagement of caregivers as key stakeholders in the management of the HIV response among the ALHIV. It will thus require strategic refocus and investment to ensure elements such as periodic engagements of caregivers and ALHIV themselves to strengthen sensitization and treatment literacy, provision of food recipes during clinic visits and transport availability. This will likely provide the necessary motivation for the adolescents and their caregivers to take a more central role in actively contributing to their health.

### Recommendations

1. We recommend the implementation of OTZ in the setting to ensure health-workers, adolescents themselves and the caregivers are all actively involved in the provision of health services.
2. Sustenance of the YAPS model but enhance the provision of family-centered care. The implementation of the integrated community service delivery model of the ministry of health needs to be scaled up to strengthen capacity of families and the wider communities in order to induce provision of optimal social support that remains critical in attaining optimal treatment outcomes among the ALHIV.
3. Whereas community-based DSDM potentially improves retention in care, it negatively affects viral load suppression rates. Therefore, we suggest that health-workers need to pay a keen attention and use continuous quality improvement approach to ensure that specific needs of the ALHIV in those models are identified and appropriately responded to.
4. Lastly, HIV programs need to continue implementing multi-month dispensing of drugs (ART), as it spurs good retention rates in care.

## Figures and Tables

**Table 1:** Basic demographics of qualitative respondents

Variables		
**Age-group**	10–14	6
	15–19	18
	20–24	8
	25–29	0
	30+	27
**Sex**	Female	31
	Male	28
**Education level**	Primary	15
	Post-primary	24
	None	20
**Occupation**	Peasant	22
	Health worker	10
	Business person	09
	Student	16
	Mechanic	1
	Disc Jockey	1

**Table 2:** Univariates for quantitative component

Variables	Frequency (N = 533)	Proportion (percentage)
**Study centres**		
TASO Mbale CoE	304	57.0
TASO Soroti CoE	229	43.0
**Current retention at 12 months**		
Active	511	95.9
Died	4	0.8
Transferred Out	8	1.5
Lost > 28days	10	1.9
**Current viral load suppression**		
Non-suppressed	134	25.1
Suppressed	399	74.9
**Viremia (for Non-suppressed, N = 134)**		
Low viremia (> 200&<1000 copies/mL)	47	34.1
High viremia ( > = 1000 copies/mL)	87	65.9
**Adherence scores**		
Good	494	92.7
Fair	27	5.1
Poor	12	2.3
**Current age**		
11–14 Years	199	37.3
15–18 Years	334	62.7
**Age at diagnosis**		
0–2 Years	232	43.5
3–5 Years	156	29.3
6–10 Years	118	22.1
11–15 Years	24	4.5
> 15 Years	3	0.6
**Sex**		
Female	289	54.2
Male	244	45.8
**Pregnancy status (N = 289)**		
Yes	2	0.7
No	287	99.3
**School going status**		
Not at school	165	31.0
At school	368	69.0
**Caregiver present**		
No	8	1.5
Yes	525	98.5
**Caregiver relationship (N = 525)**		
Biological parent	324	61.7
Guardian	201	38.3
**Caregiver HIV status (N = 525)**		
HIV Negative	153	29.1
HIV Positive	230	43.8
Unknown	142	27.0
**Baseline WHO clinical stage**		
Clinical stage I	50	9.4
Clinical stage II	438	82.2
Clinical stage III	32	6.0
Clinical stage IV	13	2.4
**Baseline CD4 count**		
< 200 copies	66	12.4
>=200 copies	209	39.2
Not done	258	48.4
**Current ART regimen**		
ABC-3TC-DTG	88	16.5
AZT-3TC-DTG	29	5.4
TDF-3TC-DTG	406	76.2
TDF-3TC-LPV/r	1	0.2
Other	9	1.7
**Current ART line**		
First line	449	84.2
Second line	71	13.3
Third line	13	2.4
**Current DSDM approach**		
CCLAD (community client-led ART Delivery)	25	4.7
CDDP (Community drug delivery points)	106	19.9
FBG (facility-based groups)	354	66.4
FBIM (facility-based individual management)	40	7.5
FTDR (Fast-track drug refills)	8	1.5
**MUAC (Mid-upper arm circumference)**		
Green	498	93.4
Yellow	23	4.3
Red	12	2.3
**TB status**		
No signs and symptoms	498	93.4
Presumptive	28	5.3
TB diagnosed	7	1.3
**OVC (orphaned and vulnerable children) status**		
Ever enrolled	323	60.6
Never enrolled	210	39.4
**Benefited from OVC services(N = 323)**		
No	62	19.2
Yes	261	80.8
**MMD (multi-month dispensing)**		
< 3_months	59	11.1
3 to 5 months	273	51.2
More than 5 months	201	37.7
**Distance to facility**		
< 5km	207	38.8
>=5km	326	61.2
**Disclosure status**		
Yes	522	97.9
No	11	2.1

**Table 3:** Bivariate analysis of association between various categorical variables and primary outcomes

Column1	Retention at 12 months			Viral Suppression		
Variables	Retained (N = 511)	Not retained (N = 22)	P-value	Supressed (N = 399)	Nonsuppressed (N = 134)	P-value
**Adherence scores**						
Good	474(96.0)	20(4.0)	0.76	100(20.2)	394(79.8)	< 0.001**
Fair	26(96.3)	1(3.7)		24(88.9)	3(11.1)	
Poor	11(91.7)	1(8.3)		10(83.3)	2(16.7)	
**Current age**						
11–14 Years	187(94.0)	12(6.0)	0.09	48(24.1)	151(75.9)	0.68
15–18 Years	324(97.0)	10(3.0)		86(25.8)	248(74.2)	
**Age at diagnosis**						
0–2 Years	219(94.4)	13(5.6)	< 0.001**	59(25.4)	173(74.6)	0.51
3–5 Years	152(97.4)	4(2.6)		41(26.3)	115(73.7)	
6–10 Years	117(99.2)	1(0.8)		31(26.3)	87(73.7)	
11–15 Years	21(87.5)	3(12.5)		3(12.5)	21(87.5)	
>15 Years	2(66.7)	1(33.3)		0(0.0)	3(100.0)	
**Sex**						
Female	279(96.5)	10(3.5)	0.40	67(23.2)	222(76.8)	0.26
Male	232(95.9)	12(4.1)		67(27.5)	177(72.5)	
**Pregnancy status (N = 289)**						
Yes	2(100.0)	0(0.0)	0.79	0(0.0)	2(100.0)	0.44
No	277(96.5)	10(3.5)		67(23.3)	220(76.7)	
**School going status**						
Not at school	163(98.8)	2(1.2)	**0.023** **	41(24.9)	124(75.2)	0.92
At school	348(94.6)	11(5.4)		93(25.3)	275(74.7)	
**Caregiver present**						
No	8(100.0)	0(0.0)	0.55	0(0.0)	8(100.0)	0.1
Yes	503(95.8)	13(4.2)		134(25.5)	391(74.5)	
**Caregiver relationship (N = 525)**						
Biological parent	317(97.8)	7(2.2)	< 0.001**	84(25.9)	240(74.1)	0.79
Guardian	186(92.5)	15(7.5)		50(24.9)	151(75.1)	
**Caregiver HIV status (N = 525)**						
HIV Negative	144(94.1)	9(5.9)	0.13	40	113	0.73
HIV Positive	225(97.8)	5(2.2)		55	175	
Unknown	134(94.4)	8(5.6)		39	103	
**Baseline WHO clinical stage**						
Clinical stage I	48(96.0)	2(4.0)	0.54	7(14.0)	43(86.0)	0.07
Clinical stage II	418(95.4)	20(4.6)		110(25.1)	328(74.9)	
Clinical stage III	32(100.0)	0(0.0)		12(37.5)	20(62.5)	
Clinical stage IV	13(100.0)	0(0.0)		5(38.5)	8(61.5)	
**Baseline CD4 count**						
< 200 copies	64(97.0)	2(3.0)	0.34	20(30.3)	46(69.7)	0.41
>=200 copies	203(97.1)	6(2.9)		55(26.3)	154(73.7)	
Not done	244(94.6)	14(5.4)		59(22.9)	199(77.1)	
**Current ART regimen**						
**Current ART line**						
First line	431(96.0)	9(4.0)	0.61	100(22.3)	349(77.7)	**0.002** **
Second line	67(94.4)	4(5.6)		29(40.9)	42(59.1)	
Third line	13(100.0)	0(0.0)		5(38.5)	8(61.5)	
**Current DSDM approach**						
CCLAD	25(100.0)	0(0.0)	0.58	5(20.0)	20(80.0)	< 0.001**
CDDP	101(95.3)	5(4.7)		35(33.0)	71(67.0)	
FBG	339(95.8)	15(4.2)		64(18.1)	290(81.9)	
FBIM	39(97.5)	1(2.5)		28(70.0)	12(30.0)	
FTDR	7(87.5)	1(12.5)		2(25.0)	6(75.0)	
**MUAC**						
Green	477(95.8)	12(4.2)	0.77	129(25.9)	369(74.1)	0.3
Yellow	22(95.7)	1(4.3)		3(13.0)	20(87.0)	
Red	12(100.0)	0(0.0)		2(16.7)	10(83.3)	
**TB status**						
No signs and symptoms	477(95.8)	12(4.2)	0.22	127(25.5)	371(74.5)	0.65
Presumptive	28(100.0)	0(0.0)		5(17.9)	23(82.1)	
TB diagnosed	6(85.7)	1(14.3)		2(28.6)	5(71.4)	
**OVC status**						
Ever enrolled	316(97.8)	7(2.2)	< 0.001**	78(24.2)	245(75.8)	0.51
Never enrolled	195(92.9)	15(7.1)		56(26.7)	154(73.3)	
**Benefited from OVC services(N = 323)**						
No	62(100.0)	0(0.0)	0.19	14(22.6)	48(77.4)	0.75
Yes	254(97.3)	7(2.7)		64(24.5)	197(75.5)	
**MMD**						
< 3_months	51(86.4)	8(13.6)	< 0.001**	25(42.4)	34(57.6)	< 0.001**
3 to 5 months	261(95.6)	12(4.4)		74(27.1)	199(72.9)	
More than 5 months	199(99.0)	2(1.0)		35(17.4)	166(82.6)	
**Distance to facility**						
< 5km	203(98.1)	4(98.1)	**0.042** **	55(26.6)	152(73.4)	0.54
>=5km	308(94.5)	18(5.5)		79(24.2)	247(75.8)	
**Disclosure status**						
Yes	502(96.2)	11(3.8)	**0.018** **	131(25.1)	391(74.9)	0.87
No	9(81.8)	2(18.2)		3(27.3)	8(72.7)	

** (Significant, P < 0.05)

**Table 4:** Logistic regression for predictors of retention

Variables	Retention at 12 months							
	Unadjusted				Adjusted			
	OR	P-value	CI		OR	P-value	CI	
**Current weight(kgs)**	1.0698	**0.006** **	1.0198	1.1222	1.1060	0.045	1.0206	1.1985
**Duration on ART**	1.0394	0.483	0.9930	1.0150	0.9861	0.306	0.9600	1.0129
**Current age**								
11–14 Years	1				1			
15–18 Years	2.0791	0.095	0.8806	4.9089	1.5005	0.584	0.3515	6.4054
**Age at diagnosis**								
0–2 Years	1				1			
3–5 Years	2.2557	0.162	0.7209	7.0578	2.8966	0.197	0.5759	14.5703
6–10 Years	6.9452	0.064	0.8957	53.8537	4.4394	0.285	0.2893	68.1150
11–15 Years	0.4155	0.197	0.1094	1.5776	0.1333	0.275	0.0036	4.9563
>15 Years	0.1187	0.090	0.0101	1.3963	0.0062	0.099	0.0000	2.6218
**School going status**								
Not at school	1				1			
At school	0.2135	**0.04** **	0.0492	0.9256	0.1488	**0.041** **	0.0240	0.9218
**Caregiver relationship (N = 525)**								
Biological parent	1				1			
Guardian	0.2738	**0.01** **	0.1095	0.6844	0.4861	0.463	0.0709	3.3353
**Caregiver current VL status**								
Suppressed	1							
Nonsuppressed	0.0478	**0.002** **	0.0069	0.3369				
**Caregiver HIV status (N = 525)**								
HIV Positive	1				1			
HIV Negative	0.3556	0.070	0.1168	1.0822	0.5669	0.614	0.0626	5.1368
Unknown HIV status	0.3722	0.090	0.1193	1.1611	0.2849	0.209	0.0402	2.0194
**OVC status**								
Ever enrolled	1				1			
Never enrolled	0.2880	**0.01** **	0.1153	0.7194	0.2625	**0.023** **	0.0830	0.8300
**Multi Month Dispensing (MMD)**								
< 3_months	1				1			
3 to 5 months	3.4118	**0.01** **	1.3268	8.7732	5.3983	**0.010** **	1.4909	19.5460
More than 5 months	15.6078	< 0.001**	3.2109	75.8672	32.6287	< 0.001**	5.1446	206.9404
**Distance to facility**								
< 5km	1				1			
>=5km	0.3372	**0.05** **	0.1124	1.0117	0.4689	0.351	0.0955	2.3017
**Disclosure status**								
Yes	1				1			
No	0.1793	**0.04** **	0.0363	0.8858	0.3610	0.386	0.0361	3.6078

** Significant (P < 0.05)

**Table 5:** Logistic regression for predictors of VLS

Variables	Viral load suppression status							
	Unadjusted				Adjusted			
	OR	P-value	CI		OR	P-value	CI	
**Adherence scores**								
Good	1				1			
Fair	0.1250	< 0.001**	0.0376	0.4156	0.066	0.002**	0.0115	0.3850
Poor	0.2000	0.04**	0.0438	0.9141	1			
**Current weight(kgs)**	1.0228	0.03**	1.0027	1.0433	1.032	0.223	0.9813	1.0843
**Duration on ART**	0.9949	0.87	0.9372	1.0564	1.012	0.060	0.9995	1.0246
**Current age**								
11–14 Years	1				1			
15–18 Years	0.9167	0.68	0.6102	1.3771	0.342	0.048**	0.1180	0.9885
**School going status**								
At school	1				1			
Not at school	1.0228	0.917	0.6692	1.5632	0.840	0.746	0.2927	2.4119
**Caregiver relationship (N = 525)**								
Biological parent	1				1			
Guardian	1.0570	0.788	0.7050	1.5847	0.851	0.893	0.0812	8.9152
**Caregiver current VL status**								
Suppressed	1				1			
Non-suppressed	0.1424	0.007**	0.0344	0.5903	0.144	0.038**	0.0232	0.8944
**Current ART line**								
First line	1				1			
Second line	0.4150	.001**	0.2459	0.7003	0.242	0.006**	0.0873	0.6724
Third line	0.4585	0.18	1.4657	1.4339	1			
**Current DSDM approach**								
FBG	1				1			
CCLAD	0.8828	0.81	0.3191	0.2442	1.098	0.908	0.2270	5.3072
CDDP	0.4477	.001**	0.2750	0.7289	2.341	0.266	0.5227	10.4851
FBIM	0.0946	< 0.001**	0.0456	0.1961	0.152	0.052	0.0226	1.0180
FTDR	0.6621	0.619	0.1304	3.3609	0.723	0.843	0.0290	18.0360
**OVC status**								
Ever enrolled	1				1			
Never enrolled	0.8755	0.513	0.5880	1.3036	0.630	0.266	0.2796	1.4209
**MMD**								
< 3_months	1				1			
3 to 5 months	1.9773	< 0.022**	1.1051	3.5380	1.666	0.490	0.3916	7.0842
More than 5 months	3.4874	< 0.0001**	1.8524	6.5656	3.095	0.171	0.6145	15.5846
**Distance to facility**								
< 5km	1				1			
>=5km	1.1313	0.545	0.7590	1.6863	0.966	0.946	0.3572	2.6140

## Data Availability

The dataset(s) supporting the conclusions of this article is(are) included within the article (and its additional file(s)).

## Abbreviations

ALHIV: adolescents living with HIV
ART: anti-retroviral therapy
CCLAD: Community client-led ART Delivery
CDDP: Community drug delivery points
DTG: Dolutegravir
DSDM: Differentiate service delivery model
FBIM: Facility-based individual management
FGD: Facility-based group
FTDR: Fast-track drug refill
MMD: multi-month dispensing of drugs
MUAC: Mid-upper arm circumference
OTZ: Operation Triple Zero
OVC: Orphaned and vulnerable children
YAPS: Youth and adolescent treatment supporter
VLS: viral load suppression
